# Thermal Calcination-Based Production of SnO_2_ Nanopowder: An Analysis of SnO_2_ Nanoparticle Characteristics and Antibacterial Activities

**DOI:** 10.3390/nano8040250

**Published:** 2018-04-17

**Authors:** Naif Mohammed Al-Hada, Halimah Mohamed Kamari, Anwar Ali Baqer, Abdul H. Shaari, Elias Saion

**Affiliations:** 1 Department of Physics, Faculty of Science, Universiti Putra Malaysia, Serdang 43400, Selangor, Malaysia; anwaralibaqerkarm@yahoo.com (A.A.B.); ahalim@upm.edu.my (A.H.S.); emansaion@gmail.com (E.S.); 2 Department of Physics, Faculty of Science for Women, University of Baghdad, Baghdad 10071, Iraq; 3 Iinstitute for Mathematical Research (INSPEM), Universiti Putra Malaysia, Serdang 43400, Selangor, Malaysia

**Keywords:** tin oxide nanoparticles, calcination method, antibacterial activity

## Abstract

SnO_2_ nanoparticle production using thermal treatment with tin(II) chloride dihydrate and polyvinylpyrrolidone capping agent precursor materials for calcination was investigated. Samples were analyzed using X-ray diffraction (XRD), Scanning Electron Microscopy (SEM), energy dispersive X-ray (EDX), transmission electron microscopy (TEM), Fourier Transform Infrared Spectroscopy (FT-IR), X-ray photoelectron spectroscopy (XPS), diffuse UV-vis reflectance spectra, photoluminescence (PL) spectra and the electron spin resonance (ESR). XRD analysis found tetragonal crystalline structures in the SnO_2_ nanoparticles generated through calcination. EDX and FT-IR spectroscopy phase analysis verified the derivation of the Sn and O in the SnO_2_ nanoparticle samples from the precursor materials. An average nanoparticle size of 4–15.5 nm was achieved by increasing calcination temperature from 500 °C to 800 °C, as confirmed through TEM. The valence state and surface composition of the resulting nanoparticle were analyzed using XPS. Diffuse UV-vis reflectance spectra were used to evaluate the optical energy gap using the Kubelka-Munk equation. Greater calcination temperature resulted in the energy band gap falling from 3.90 eV to 3.64 eV. PL spectra indicated a positive relationship between particle size and photoluminescence. Magnetic features were investigated through ESR, which revealed the presence of unpaired electrons. The magnetic field resonance decreases along with an increase of the *g*-factor value as the calcination temperature increased from 500 °C to 800 °C. Finally, *Escherichia coli ATCC 25922 Gram* (–*ve*) and *Bacillus subtilis UPMC 1175 Gram* (+*ve*) were used for in vitro evaluation of the tin oxide nanoparticle’s antibacterial activity. This work indicated that the zone of inhibition of 22 mm has good antibacterial activity toward the Gram-positive *B. subtilis UPMC 1175*.

## 1. Introduction

Nanoscience is playing a major role in the research and development of devices, nanoplatforms, systems and structures across numerous areas [1,2,3], with an increasing number of studies exploring the utilization of functionalized, biodegradable and biocompatible nonmaterial [3,4]. Due to its specific attributes, tin oxide (SnO_2_) has been applied as a semiconductor nanomaterial in several different studies. Tin oxide (or cassiterite) is classified as a II–VI composite semiconductor on account of its Groups II and VI Periodic Table components, with 3.60 eV and 3.75 eV direct and indirect energy band gaps [5,6,7,8,9]. The structural attributes of nanoparticles provide numerous benefits across a great number of applications [10,11,12], with a unique tetragonal crystalline structure and metal *n*-type oxide demonstrating 3.8 eV and 4.1 eV direct and indirect energy band gaps [13].

Given the above points, SnO_2_ nanomaterials can be used with positive effect in a range of applications [14,15,16], with the pellucidity shown in the observable solar spectrum having been utilized in optoelectronic devices such as solar photovoltaics [17,18,19]. Other attributes of the SnO_2_ nanostructure have been utilized for diodes, gas sensors, catalysis, antibacterial activities, and biomedical purposes [12,20,21,22,23,24,25,26,27].

SnO_2_ nanostructure preparation can be achieved using several different techniques. These include the co-precipitation [26], sol-gel [28,29], solvothermal decomposition [30,31], microwave hydrothermal synthesis [32,33] and precipitation [34], techniques, as well as hydrothermal synthesis [35,36], and the polyol [37], solvothermal [38], and by microwave [39,40,41] methods. However, issues related to the complexity of the synthetic process, including the creation of effluent by-product, reagent toxicity, and longer reaction times, have made industrial SnO_2_ nanopowder generation difficult to achieve, with the creation of a pure powder product having been entirely unachievable through the aforementioned techniques [42]. A deep literature on the currently investigated research topic has been reviewed. There has been no published research study to come up with such a thermal treatment of SnO_2_ nanoparticles related tandem analysis of antibacterial activity.

The novelty of the present method is to introduce several benefits e.g., flexible, easy to handle and permits reproducible quality. It enables the production of nanoparticles with the desired properties because it can regulate particle size with high purity nanoparticles. In addition, it makes a limited use of chemicals with non-toxic effluences into the drainage system and therefore it does not cause damage to the environment [43,44,45,46,47,48,49,50,51]. Furthermore, it has the potential product to be employed on a large scale in industrial and biological applications. This study addresses the gap in the literature by using basic thermal treatment techniques to produce an SnO_2_ nanopowder without waste production, which has environmental advantages [52,53].

This paper discusses and explores the use of a novel thermal technique, wherein a precursor chloride metallic ion is used with a polyvinylpyrrolidone (PVP) capping agent before calcination for SnO_2_ nanoparticle synthesis and the production of a pure nanopowder. This study also analyzes the impact of temperature changes on the SnO_2_ nanopowder’s morphological, structural, and optical characteristics. The structure of the resulting product is investigated using various analyses, with antimicrobial activity also analyzed.

## 2. Materials and Methods

### 2.1. Materials

No additional purification treatment was applied to any of the materials prior to the experiment, with all materials being standard research-grade chemicals provided by Sigma-Aldrich, St. Louis, MO, USA. The specific materials included tin(II) chloride dihydrate SnCl_2_·2H_2_O (M_W_ = 225.65 g/mol), 0.1 HCl, polyvinylpyrrolidone (PVP) (C_6_H_9_NO)*_n_* (Mw = 58,000 g/mol), and deionized water.

### 2.2. Methodology

The initial solutions were created with 4 g of PVP in 100 mL of deionized water, with the solutions then incubated at 70 °C while being vigorously stirred for 2 h. At the 2 h mark, 0.2 mmol of tin(II) chloride dihydrate was introduced and mixed into the solution. The resulting solutions were poured using a glass plate before being placed in the oven at a temperature of 80 °C for 24 h. The dried solution was then ground into a powder using a pestle and mortar for 30 min. Calcination at various temperatures was then conducted in a box furnace over the course of 3 h, producing the tin oxide nanoparticles for analysis. 

### 2.3. Analysis of Nanoparticle Characteristics

Several methods were used to characterize the tin oxide nanoparticles’ morphological, structural, and optical attributes. For structural characterization, X-ray diffractometer (XRD Shimadzu 6000, Lelyweg1, Almelo, The Netherlands) was used with Cu Kα radiation at a 0.154 nm wavelength at the 2θ range of 4° and 80°. Fourier-transform infrared (FT-IR) spectroscopy analysis was also performed at the 280–4000 cm^−1^ range. An accelerated voltage of 200 kV was used to obtain transmission electron microscopy (TEM) images, with an energy-dispersive X-ray (EDX) spectrometer (7533, Oxford Instruments, Oxford, UK) used to conduct EDX spectroscopy. The X-ray source was obtained using monochromatic Al-K_α_ (*hv* = 1486.6 eV) at 25.6 W with a 100 µm beam diameter for X-ray photoelectron spectroscopy (XPS) (ULVAC-PHI Quantera II, Ulvac-PHI, Inc., Tokyo, Japan). Additionally, wide scan analysis was conducted with a pass energy of 280 eV with 1 eV for each step, with narrow scan/chemical states analysis carried out with a pass energy of 112 eV with 0.1 eV for each step. Modification of binding energies C–C and C–H to 284.8 eV was performed to correct the charge at C 1s before deconvolution. The optical attributes of the nanoparticle were analyzed using a UV-vis spectrophotometer (Shimadzu UV-3600, Kyoto, Japan) at a 200–800 nm wavelength at room temperature. Photoluminescence (PL) was analyzed using Perkin Elmer spectrofluorometer LS-55, Waltham, MA, USA with a Xenon lamp at room temperature. Finally, analysis of colony-forming units was conducted using an agar plate after incubation to assess the antimicrobial activity of the SnO_2_ nanoparticle.

### 2.4. Examination of Antibacterial Activity

An in vitro antimicrobial analysis was performed using the disc diffusion technique to explore the antibiotic capabilities of the SnO_2_ nanoparticles in the event of bacterial attack. This allows for a comparison of the resistance to antimicrobial compounds. The prepared nanoparticles were also tested with *Escherichia coli ATCC 25922 Gram* (−*ve*) and *Bacillus subtilis UPMC 1175 Gram* (*+ve*)*.* Here, a paper disc with a 6 mm diameter was suspended in 100 mg of each nanoparticle with 10 mL of deionized water before being subjected to incubation for drying. Microbe growth was then permitted by placing papers onto plates, with a 10^8^-cell standardized microbe culture in accordance with the 0.5 McFarland standard. The plates were subject to 30–37 °C inversion, with a 48-h incubation period to encourage bacteria growth. Following incubation, the plates were analyzed with inhibition zone diameters recorded (mm). Each test went through three iterations with the average value taken as the result. Muller Hinton agar media has been used as nutrient, *Streptomycin* (100 mg/mL) standard and distilled water have been used for each bacterium as a positive and as negative controls, respectively. In addition, the antibacterial test for bulk tin oxide has been compared with prepared tin oxide nanoparticles.

## 3. Results and Discussion

### 3.1. Mechanism of Nanoparticles

**Figure 1:**
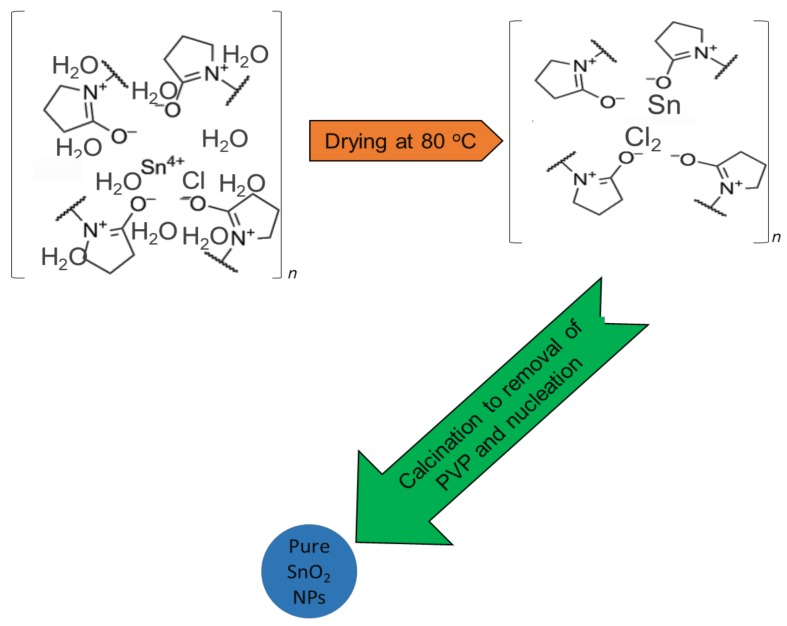
Nanoparticle growth mechanism.

Nanoparticle growth, behavior and mechanisms during calcination are illustrated in Figure 1. PVP was originally applied to complex metallic salts as a stability agent, and it is frequently used with amide groups of pyrrolidone rings as well as methylene groups for steric and electrostatic stabilization. Metallic ions were suppressed during the mixing stage to capture them with the amine group through ionic-dipole interaction within the polymeric chains. Due to the removal of H_2_O during the drying process, the metallic cations become immobile inside the polymer cavity. Organic materials are converted into gases (e.g., N_2_, NO, CO, CO_2_) during calcination (from 500 °C to 800 °C), at which point PVP also influences the tin oxide nanoparticle nuclei formation. The Ostwald ripening phenomenon would arise without PVP, causing the nanoparticle to expand and demonstrate greater surface energy. In this case, steric hindrance has been deactivated by PVP, preventing the accumulation of nanoparticles [54,55]. Therefore, the use of PVP as a preventative agent in the breakdown of the metal ions on the nanoparticle surface renders PVP an effective technique for minimizing nanoparticle gain [56,57,58].

### 3.2. XRD Analysis Structural Analysis

**Figure 2:**
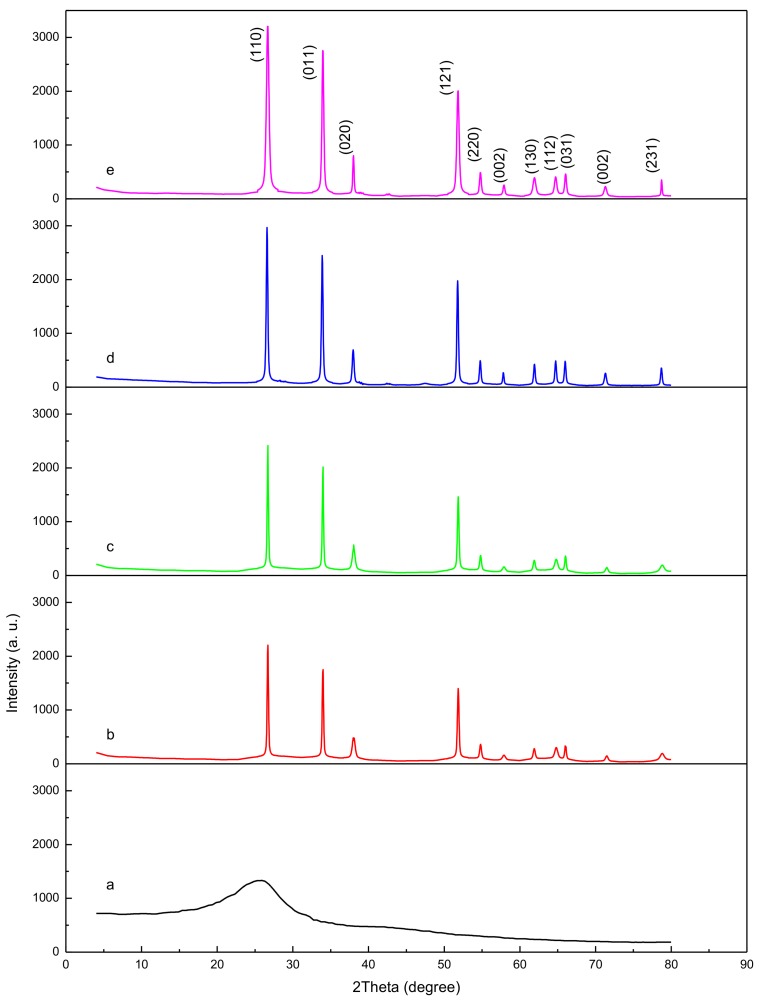
Nanoparticle XRD patterns prepared at 30 °C/room temperature (**a**). with calcination at 500 °C (**b**), 600 °C (**c**), 700 °C (**d**), and 800 °C (**e**).

The results of the XRD analysis are illustrated in Figure 2, demonstrating the pre- and post-calcination attributes of the SnO_2_ nanoparticles. As illustrated in Figure 2a, the broad spectrum of the pre-calcination nanoparticles indicates the product’s amorphous-like behavior, as well as the non-development of the nanoparticles. The peaks shown in Figure 2b–e, on the other hand, demonstrate nanoparticle formation following calcination at 500 °C. The figures further illustrate the positive association between calcination and the height, sharpness, and narrowness of the peaks, indicating that the nanoparticles crystalize as the temperature increases. As illustrated in the TEM images, this is due to the gradual increase of the crystalline volume to surface ratio due to the growth in particle size. The correlation between temperature and crystalline size is illustrated in Table 1. 

Crystallite size was analyzed using Bragg’s law. The SnO_2_ nanoparticles were found to have a standard tetragonal structure (JCPDS 00-041-1445) [59,60], based on the diffraction peaks (110), (011), (020), (121), (220), (002), (130), (112), (031), (022) and (231) in the diffraction patterns. A range of 3–14 mm in crystallite size was determined for peak (110) (the peak with the greatest intensity) based on the following Scherrer equation:

*D* = *K*λ/βcosθ (1)

where *K* is the Scherrer constant (0.9), λ is the X-ray irradiation wavelength (1.5418 Å), and β is the XRD peak width (FWHM).

### 3.3. SEM Analysis

**Figure 3:**
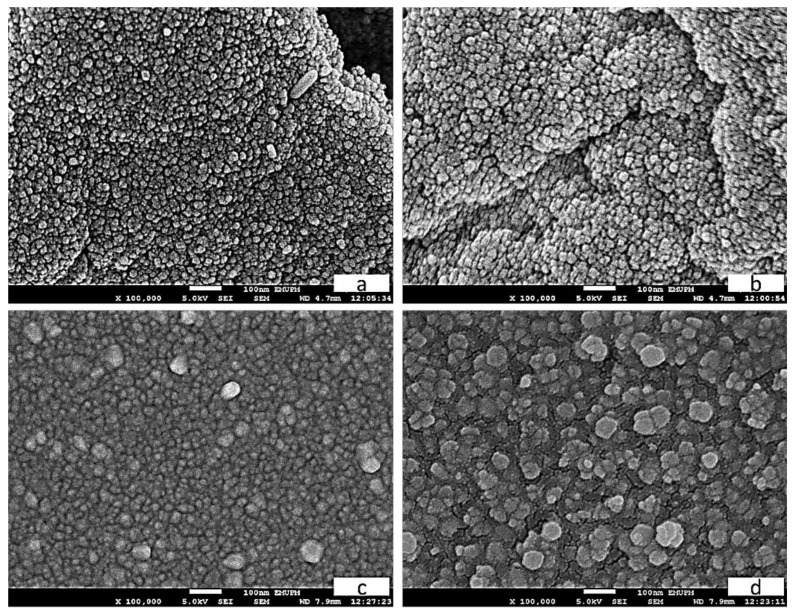
Nanoparticle SEM images at calcining temperatures of 500 °C (**a**), 600 °C (**b**), 700 °C (**c**) and 800 °C (**d**).

The tin oxide nanoparticles’ surface morphology was analyzed using SEM, with the micrographs for each calcination temperature illustrated in Figure 3. As shown, the structure of the nanoparticles is grain-shaped and spherical, which echoes the results of previous work [61,62]. It is evident from the analysis that the nanoparticles take the shape of a large, near-spherical grain with regularities at high temperatures (Figure 3a,b). It appears that temperatures of 500–800 °C during calcination lead to greater nucleation and growth rates, as shown in Figure 3b–d. 

### 3.4. EDX Spectrum Analysis

**Figure 4:**
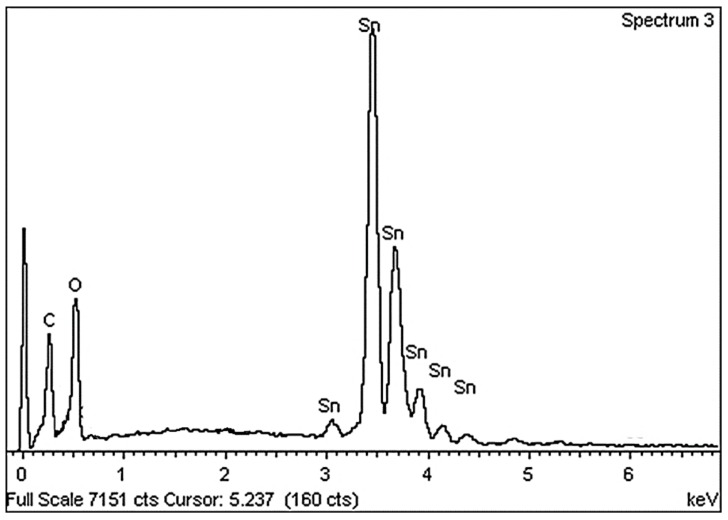
Nanoparticle EDX spectrum at 600 °C calcination.

To analyze nanoparticle composition at various calcination temperatures EDX spectroscopy was conducted on the sample, with the results presented in Figure 4. Here, Sn: O peaks indicate the existence of Sn: O elements, with Table 2 illustrating their atomic percentages. The peak of 0.3 keV represents the carbon film holder used for the analysis. The results confirm that the final nanopowder is comprised of pure tin oxide, with the Sn: O atomic percentage only determined in the final sample.

### 3.5. TEM Analysis

**Figure 5:**
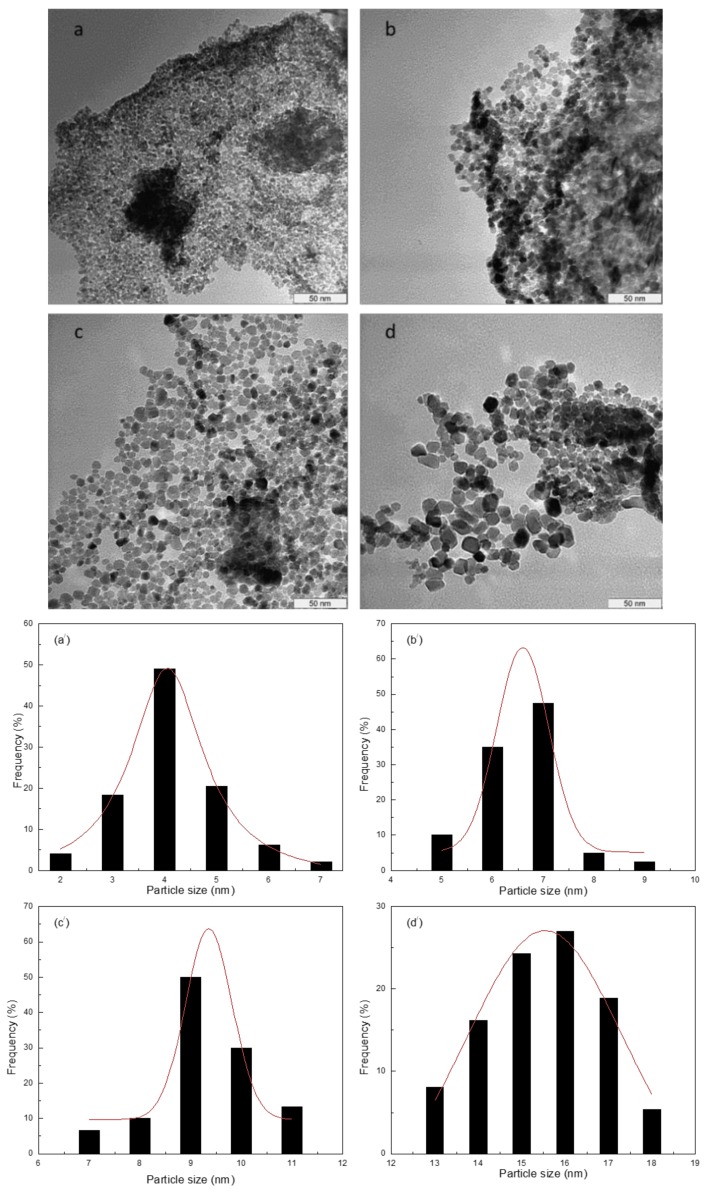
Nanoparticle size distribution histogram and TEM micrographs at calcination temperatures of 500 °C (**a**,**a’**) 600 °C (**b**,**b’**) 700 °C (**c**,**c’**) and 800 °C (**d**,**d’**).

Nanoparticle microstructure analysis was performed using the TEM technique. As illustrated in Figure 5, the nanoparticles were found to have a regular morphology, with nanoparticle characteristics then analyzed at various calcination temperatures (Figure 5a–d). The results confirm that the product morphology is spherical and homogeneous. The correlation between particle size and calcination temperature, as noted previously, is caused by the accrual of nearby particles as the surface melts at the highest temperatures [63,64,65]. The use of this method for nanoparticle formation has been shown to be effective, with PVP impacting particle size due to its suppressive function on particle accumulation. Thus, PVP is shown to be a stabilizer of particle size, supporting nucleation, particle size growth, and particle regularity. PVP has been used previously to control particle size and prevent nanoparticle accretion [55,56,57,58,66,67,68]. The results illustrated in Table 1 allow for a comparison of the XRD and TEM analyses, further highlighting the relationship between calcination temperature and particle size (4–15.5 mm). 

### 3.6. Functional Analysis

**Figure 6:**
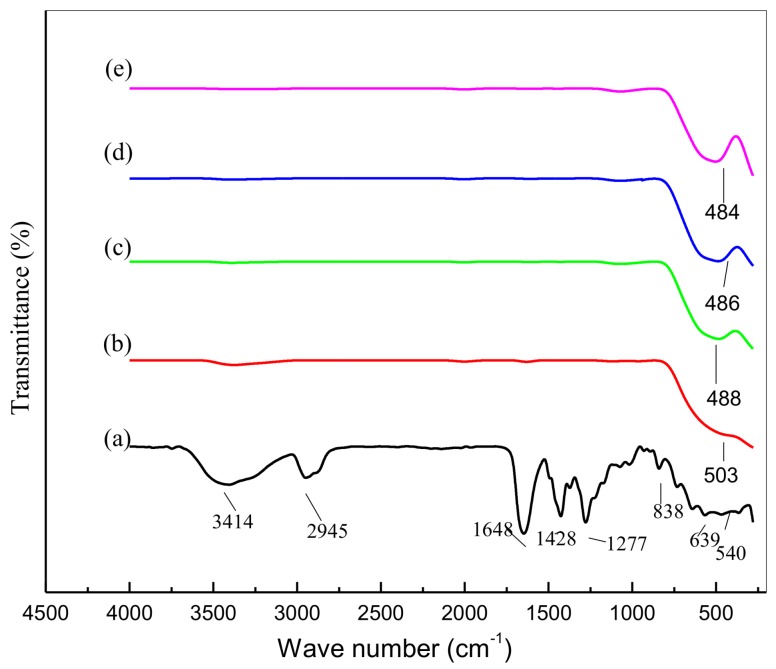
Nanoparticle FTIR spectra at the 280–4500 cm^−1^ range at room temperature (**a**) 500 °C (**b**), 600 °C (**c**), 700 °C (**d**), and 800 °C (**e**).

Figure 6 illustrates the FTIR spectrum at 280–4000 cm^−1^ for the thermal calcination-generated SnO_2_ nanoparticles. The nanoparticles and organic compounds are reflected in the absorption peaks shown in Figure 6a, with wave numbers of 3414 cm^−1^ assigned to N–H stretching vibration, 2945 cm^−1^ to C–H and 1646 cm^−1^ to C=O. The 1428 cm^−1^ absorption peak was assigned to the C–H bending vibration (methylene group), with the 1277 cm^−1^ peak assigned to the C–N stretching vibration [64]. The 839 cm^−1^ absorption peak relates to the C–C ring vibration, the 639 cm^−1^ peaks to the C–N=O bending vibration [63], and the 540 cm^−1^ to the O–Sn–O vibration. It is believed that the purity of the tin oxide nanoparticles is the cause of the single absorption peak, as well as the SnO_2_ nanoparticle wave number fluctuation based on calcination temperature. The enhanced crystallinity of the nanoparticles supports this point regarding the impact of calcination. The sharper peaks, representing increased calcination, are illustrated in Figure 6, with these peaks suggesting that the tin oxide product becomes more crystalline in structure because of increased calcination. 

### 3.7. Compositional Analysis

**Figure 7:**
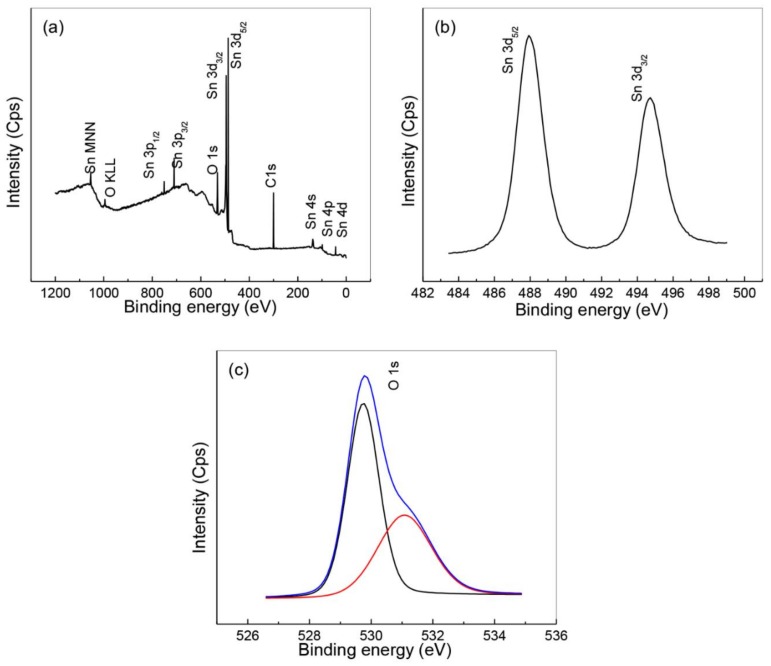
Nanoparticle XPS spectra for survey (**a**), tin (**b**), and oxygen (**c**).

The Sn and O elements’ compositions phase and chemical state were analyzed using X-ray photoelectron spectroscopy (XPS). The existence of Sn, O and C elements is confirmed in the XPS results shown in Figure 7a, with high-res XPS spectra for Sn 3d_3/2_ and Sn 3d_5/2_ shown in Figure 7b. The binding energies of 487.8 eV for Sn 3d_5/2_ peak and 494.9 eV for the Sn 3d_3/2_ peak are in line with the results presented in earlier research [19,27,69]. The deconvoluted O 1s spectrum demonstrates binding energies of 529.7 eV and 531.1 eV for two forms of oxygen (Figure 7c), which have been found to be correlated with SnO_2_ [70,71]. The results appear to verify the purity of the nanoparticle elements’ oxidation states. 

### 3.8. UV-Vis Diffuse Reflectance Spectra (DRS) Analysis

**Figure 8:**
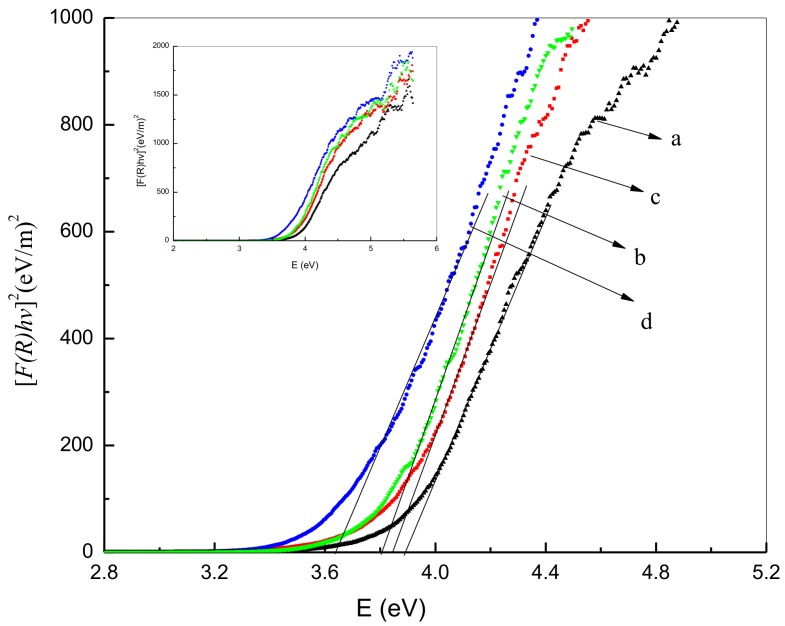
Nanoparticle energy band gap at calcination temperatures of 500 °C (**a**), 600 °C (**b**), 700 °C (**c**), and 800 °C (**d**).

To identify the nanoparticles’ energy band gaps from diffuse reflectance spectra for nanoparticle samples produced at various calcination temperatures, the Kubelka-Munk method was used. This entailed plotting the square of the Kubelka-Munk function F(R)^2^ vs. energy and extending the linear part of the curve to F(R)^2^ = 0. This is demonstrated in Figure 8a–d with the process denoting the generation of tin oxide nanoparticles’ direct band gap energy. The results indicate that an increase in calcination temperature is associated with a decrease in energy band gap value, likely due to a quantum size effect. It is suggested that the decrease in band gap could be due to transitions between Sn^2+^ ion d-shell electrons’ valance and conduction bands [72]. Given this, it is difficult to eradicate the particle size effect on the band gap. It is possible to change the band structure and material attributes due to the reduction in the size of the particle with a reduction in band gap similarly resulting in greater particle size. Thus, the presence of smaller particle sizes can be associated with overlap, with s-electron and p-electron conduction bands separating in higher energy conditions. Research indicates low nuclear potential for electron conduction at a Fermi level distance far from the center of the particle, meaning that absorption energy and conduction band energy will be the same in the case of transitions within the chosen quantum numbers. Higher calcination temperatures were associated with lower band gap values (Table 1), and it is suggested that the rise in temperature may cause an incremental increase in the absorption coefficient because of an increase of defected states. Electron-hole pairs are produced through photon absorption, generating a field that could change the optical attributes and electronic structure of nanoparticle products. 

### 3.9. Photoluminescence Studies

**Figure 9:**
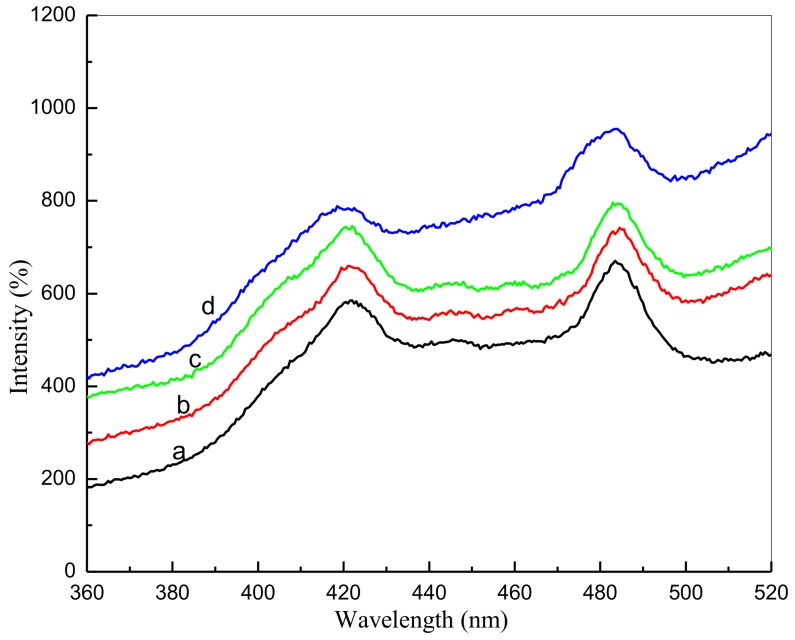
Nanoparticle photoluminescence at calcination temperatures of 500 °C (**a**), 600 °C (**b**), 700 °C (**c**), and 800 °C (**d**).

Room temperature nanoparticle photoluminescence (PL) spectra were acquired as part of the analyses with Figure 9 demonstrating a broad emission scattering of approximately 425 nm to 487 nm for the PVP-prepared tin oxide nanoparticles. This is believed to be due to the compounded effect and energy states within the valence and conduction bands [73]. The first peak is believed to be caused by the recombination of electron-hole pairs in oxygen and metal vacancies, with the second peak being evident in the PL spectra of the nanoparticles transitioning between valence and conduction bands [74,75]. As the calcination temperature increases, intensification of the PL is demonstrated, with the greatest intensity shown at a temperature of 800 °C (also the point of ultimate crystallinity). 

### 3.10. Electron Spin Resonance

**Figure 10:**
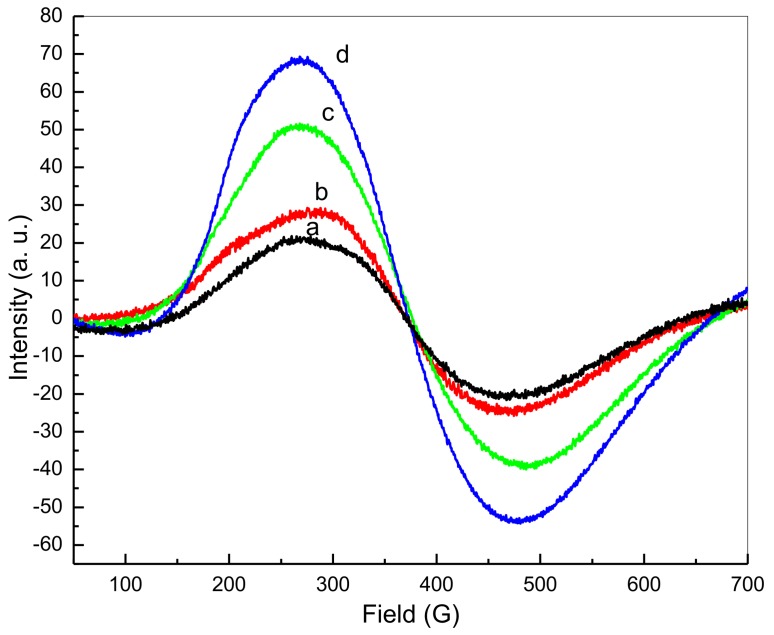
Nanoparticle ESR spectra at calcination temperatures of 500 °C (**a**), 600 °C (**b**), 700 °C (**c**), and 800 °C (**d**).

Figure 10 illustrates the electron spin resonance (ESR) spectrum for different calcination temperatures, with symmetry and a broad scope demonstrated. This is due to the presence of unpaired transition Sn^2+^ ion conduction electrons, which were present across all samples at all temperatures, indicating paramagnetic attributes. Between 377.183 G and 370.590 G, the overall resonant magnetic field appeared to decrease, with calcination temperatures increasing from 500 °C to 800 °C (Table 3). Different calcination temperatures were also found to be associated with a *g*-factor increase from 1.7395 to 1.7703, demonstrating that the increase in temperatures occurred in line with a rise in the inner-magnetic field. Thus, the results suggest that an increase in particle size results in increased magnetic attributes. The following equation can be used to express the *g*-factor value [76]:

g = (*hv*)/(β*Hr*) (2)

where *h* is Planck’s constant, *v* is microwave frequency, β is the Bohr magneton (9.274 × 10^−24^ J/T), and *Hr* is the resonant magnetic field. It is anticipated that an increase in *g*-factor will result in a decrease in the resonant magnetic field (*Hr*). This being said, the EPR spectroscopy results suggest that *v* values are constants. Previous research on SnO_2_ nanoparticles has also confirmed this increase in *g*-factor, decrease in *Hr*, and the resulting increase in magnetization.

### 3.11. Antibacterial Activity

**Figure 11:**
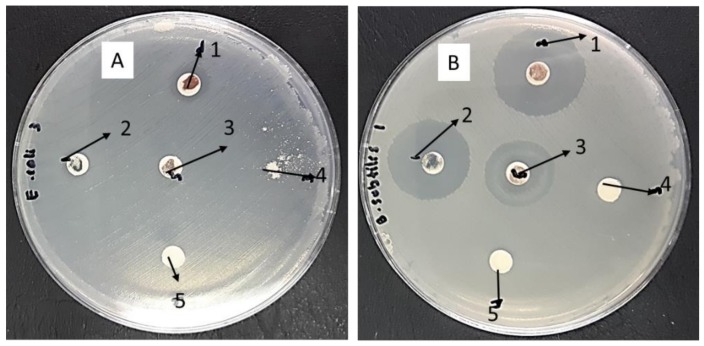
Inhibition zone test for *Escherichia coli ATCC 25922 Gram* (−*ve*) (**A**) and *Bacillus subtilis UPMC 1175 Gram* (+*ve*) (**B**), the nanoparticle samples (1 at 500 °C, 2 at 600 °C, 3 at 700 °C, and 4 at 800 °C), with lower activity observed in the bulk suspensions (5).

**Figure 12:**
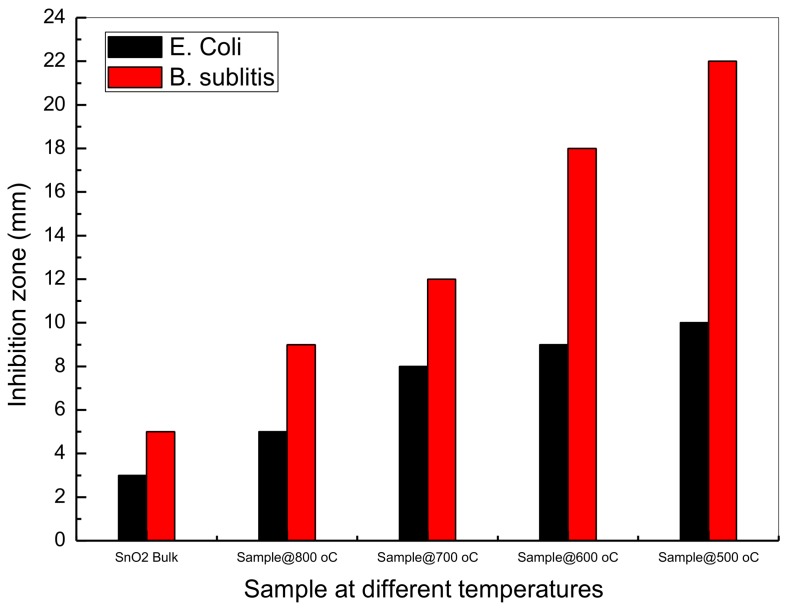
Average inhibition zone graph for SnO_2_ bulk and SnO_2_ nanoparticles against *Escherichia coli ATCC 25922 Gram* (−*ve*) and *Bacillus subtilis UPMC 1175 Gram* (+*ve*)*.*

The bulk SnO_2_ nanoparticles and SnO_2_ nanoparticles were tested for antimicrobial activity in relation to *Escherichia coli ATCC 25922 Gram* (−*ve*) and *Bacillus subtilis UPMC 1175 Gram* (+*ve*) (Figure 11 and Figure 12), with inhibition zone diameters used with the agar plates shown in millimeters. Higher activity was observed in the nanoparticle samples (1 at 500 °C, 2 at 600 °C, 3 at 700 °C, and 4 at 800 °C), with lower activity observed in the bulk suspensions (5).

The higher antimicrobial activity in the nanoparticle samples can be explained in several ways. The first mechanism could be particle size, with size being found to decrease as antimicrobial activity increases [53]. Secondly, it is suggested that antimicrobial activity may be significantly impacted by Sn^2+^ ions [77]. The increase in antimicrobial activity resulting from either mechanism has been found to be effective in curtailing *E. coli E266 N B. subtilis B29 N S*. Furthermore, research indicates that even after peroxide treatment, which is believed to interrupt antimicrobial activity, the highly bactericidal SnO_2_ nanoparticles have been found to maintain their antimicrobial effectiveness [78,79]. The reason for this is believed to be the metal oxide nanoparticles covering the perimeter of the bacterial surface membrane. It should also be highlighted that research in cell inhibition has more commonly focused on SnO_2_ nanoparticles than on the use of traditional antibiotics, suggesting that many researchers may explore biomedical applications of nanoparticles over the coming years. 

## 4. Conclusions

The results of this study demonstrate the effectiveness of thermal calcination to produce tin oxide nanoparticles. Here, the results of the XRD analysis confirmed the formation of tetragonal crystalline nanoparticles at several different calcination temperatures, with temperature also being found to increase particle size (ranging from 4 nm to 16 nm between 500 °C and 800 °C, respectively). Grain and regular morphology was identified through the conduction of SEM analysis, with EDX analysis also confirming the existence of Sn and O atoms following calcination. Their vibrational characteristics were captured based on FT-IR spectra. UV-vis absorption spectrophotometry analysis also revealed an inverse relationship between calcination temperature and optical bandgap, with a positive correlation found between particle size and photoluminescence intensity, as per the PL spectra. The results support the value of SnO_2_ application in the field of solar cell technology, with the absorption of specific solar energy wavelengths explored. 

Finally, antimicrobial activity was tested against both *Escherichia coli ATCC 25922 Gram* (−*ve*) and *Bacillus subtilis UPMC 1175 Gram* (+*ve*), with the results of the analysis indicating that the SnO_2_ nanoparticle achieves effective antimicrobial action against *Bacillus subtilis UPMC 1175 Gram* (+*ve*) at a 22 mm inhibition zone. The robustness of the methodology adopted in this study has been verified by several additional tests, with the chosen model shown to have much value in further nanotechnology and biomedical research. 

## Figures and Tables

**Table 1:** XRD, TEM and energy band gap results for tin oxide nanoparticles at different temperature.

Temperature (°C)	Crystallite Size (nm)	Particle Size (nm)	Band Gab (E_g_)
500	3	4 ± 2	3.90
600	5	6.7 ± 3	3.84
700	8	9.5 ± 2	3.80
800	14	15.5 ± 4	3.64

**Table 2:** EDX spectra showing the atomic percentages of Sn and oxygen species.

Spectrum	In Stats.	O	C	Sn	Total
Spectrum 1	Yes	60.43	4.00	35.57	100.00
Spectrum 2	Yes	66.59		33.41	100.00
Spectrum 3	Yes	64.40		35.60	100.00

**Table 3:** Magnetic parameters of SnO_2_ nanoparticles observed for ESR analysis.

Temperature (°C)	*g*-Factor	*Hr* (Oe)
500	1.7395	377.183
600	1.7519	374.497
700	1.7623	372.251
800	1.7703	370.590
